# Characterization of a Core Fungal Community and Captivity‐Induced Gut “Mycobiome” Change in Fowler's Toad ( *Anaxyrus fowleri* )

**DOI:** 10.1002/ece3.73430

**Published:** 2026-04-08

**Authors:** Alexander J. Bradshaw, Sinlan Poo, Tracey E. Malter, Rylie M. Strasbaugh, Brianna Bodner, Madison R. Hincher, Anne Devan‐Song, Javier F. Tabima

**Affiliations:** ^1^ Biology Department Clark University Worcester Massachusetts USA; ^2^ Conservation and Research Department Memphis Zoo Memphis Tennessee USA; ^3^ Department of Biological Sciences Arkansas State University Jonesboro Arkansas USA; ^4^ Conservation and Experiential Programming Department, Forest Preserves of Cook County Cook County Illinois USA; ^5^ Datascape Labs LLC Salem Oregon USA

**Keywords:** amphibians, *Anaxyrus fowleri*, conservation, *ex‐situ* husbandry, fungi, Mycobiome

## Abstract

Amphibious animals, such as frogs, are found at the intersection of aquatic and terrestrial ecosystems. They may serve as keystone and sentinel species and play key roles in nutrient cycling and food webs. In recent decades, amphibians have experienced drastic population declines due to habitat loss, climate change, and disease. These declines have prompted investments in *ex situ* conservation and captive breeding programs, which aim to reduce extinction risk by creating assurance colonies and reintroducing individuals once threats are mitigated. A critical component of these programs is proper husbandry, which ensures the health and longevity of captive populations and their ability to produce offspring that can be reintroduced into the wild. The artificial environment in captivity can profoundly impact animal behavior and health, particularly in relation to diet and nutrition. Diet not only provides nutrients and energy but also shapes the host's gut microbial community, which in turn impacts digestive health. Complex microbial communities, collectively known as the microbiome, are characterized by a high diversity of prokaryotes, microscopic fungi, and viruses. The diet‐associated microbiome is increasingly studied for its role in captive animal health and behavior, although research has focused more on bacteria than fungal communities, or the “mycobiome”. Here, we investigated the core mycobiome using metabarcoding of fungal communities in 15 wild‐caught *Anaxyrus fowleri* (Fowler's Toad), documenting shifts as toads transitioned from wild to captive settings. We identified a core set of fungal taxa and observed distinct changes in non‐core fungi associated with dietary differences associated with captivity. The non‐core mycobiome exhibited an ecological guild functional shift of the saprotrophic dominance relative to wild individuals, indicating large losses in both mycobiome diversity and functionality. These findings highlight the dynamic nature of the amphibian mycobiome and the dramatic impact captivity can have on microbial composition, providing a framework for understanding the role of the amphibian mycobiome in future conservation efforts.

## Graphical abstract

Amphibians like Fowler's Toad are vital to ecosystems but face population declines, prompting conservation efforts through captive breeding programs. This study examined how captivity and diet influence the fungal gut microbiome (mycobiome) in wild‐caught toads using metabarcoding. Results revealed a core set of fungal taxa and notable shifts in non‐core fungi, underscoring the importance of microbiome dynamics in amphibian health and conservation.

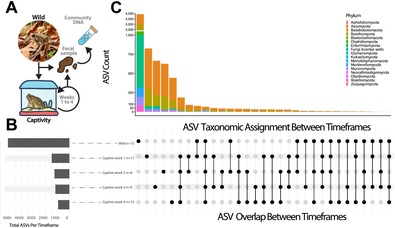

## 1 Introduction

Amphibians play a crucial role in maintaining ecosystem balance, serving as both predators and prey within aquatic and terrestrial food webs and acting as indicators of ecosystem health (Kiesecker et al. 2004). However, global populations of amphibians are experiencing drastic declines (Alroy 2015; Luedtke et al. 2023), with estimates suggesting extinction rates exceed more than 200 times the natural background levels (Blaustein et al. 2011) and exceed fossil record extinction by orders of magnitude (Alroy 2015; Tietje and Rödel 2018). These declines result from habitat destruction, climate change, pollution, and the emergence of fungal infectious diseases, such as chytridiomycosis (Alford et al. 2001; Lips 2016). While some amphibian species remain abundant, nearly 40% are now classified as threatened (Luedtke et al. 2023) by the International Union for Conservation of Nature (IUCN), making them the most threatened vertebrate groups. Conservation efforts, including in situ strategies for habitat protection and restoration (Burrow and Lance 2022) and *ex situ* captive efforts for establishing assurance colonies, breeding, and reintroduction efforts (Karlsdóttir et al. 2021), have been increasingly prioritized in response to these declines. As *ex situ* conservation strategies are implemented, increasing attention is being paid to the physiological effects of diet and the changing microbiome on animals in captivity (Trevelline et al. 2019). Captive rearing presents a particularly striking example of environmental disruption, where animals experience intense significant dietary and habitat modifications. Bringing wild animals into captivity presents several challenges, as the captive environment can be significantly different from what the animals are evolutionarily adapted to and often lacks the cues, resources, and complexity of a natural environment (Mason 2010). These differences in environment can result in variations in behavior (Kelly et al. 2025), morphology (O'Regan and Kitchener 2005; Zack et al. 2022), physiology (Turko et al. 2023), emerging infections (Taylor et al. 1999), and untold other aspects of captive animals compared to their wild counterparts. While some species can adapt to captive environments, others respond negatively to the loss of these natural cues (Mason 2010). Moreover, captive effects can be compounded throughout multiple generations (Elsbeth McPhee 2004). The challenges are reversed when a captive‐bred or captive‐reared animal is released or returned to the wild. While there are known differences between wild and captive animals, many aspects of this environmental change are yet to be explored (Turko et al. 2023). In particular, there is a paucity of information regarding the transition wild animals undergo when they are brought into captivity, and, similarly, the transition that captive animals experience when they are released into natural habitats as part of reintroduction or recovery efforts. One major significant difference between captive and wild environments is the complexity and variety of food items available to them. While animals in the wild generally consume a wide range of food types, captive animals are often limited to only a few food choices (Turko et al. 2023), with supplemental nutrients added to balance out their diets as needed. The reduction or restriction in diet can affect various aspects of an animal's development (Mitchell et al. 2021), physiology, gut microbiota (McKenzie et al. 2017; Dallas and Warne 2023), and behavior (Mason 2010; Kelleher et al. 2019, 2022). In particular, major gaps remain in understanding how captivity affects amphibian health (Lisboa et al. 2025) and microbiome stability (Kueneman et al. 2022; Korpita et al. 2023).

Microbial communities play essential roles as symbionts across the trophic structure of the natural environment. Microbial communities within the gastrointestinal (GI) tract can be incredibly diverse, including representation across all domains of life, and interact dynamically with the host (Berg et al. 2020). The microbiome has been shown to influence the overall health of a host, including dietary preferences and even neurobiological development (Sharon et al. 2016; Trevelline and Kohl 2022). However, the composition of these microbial communities is not static. The GI‐associated microbiome can shift in response to dietary alterations and environmental changes, with potentially drastic consequences for host health and adaptation (Diaz and Reese 2021).

Early studies suggest that mycobiomes play pivotal roles in host metabolism, the degradation of complex carbohydrates, pathogen resistance, immune system regulation, and also synergistically aid in the spread of both micro and macro fungi (Wagg et al. 2019; Harrison et al. 2020; Bradshaw et al. 2022; Weinstein et al. 2022). However, fungal diversity directly influences these functions, making it crucial to characterize and compare fungal communities across environmental gradients. This knowledge gap is particularly concerning in the case of amphibians, which are known to interact with fungi, producing divergent health outcomes in host populations. For instance, the chytrid fungus *Batrachochytrium dendrobatidis* (Bd) is tolerated by some amphibians (Woodhams et al. 2011; Peterson and McKenzie 2014), but has led to the severe decline or loss of other amphibian species (Longcore et al. 1999; Fisher et al. 2009; van Rooij et al. 2015; Scheele et al. 2019; Li et al. 2021).

While it is essential to examine how conditions could affect threatened and endangered targeted organisms, direct experimental manipulation is often not possible due to their protected status, small population size, and the high risks it poses in disrupting breeding and recovery plans. However, one way to remedy this is by utilizing non‐endangered species as a trial for future work. Here we chose Fowler's Toad ( *Anaxyrus fowleri* ), which is abundant and widespread, native to the eastern and southeastern United States, with ranges extending northward into parts of southern Canada (Harding 1997; Conant and Collins 1998). Ecologically, Fowler's Toads occupy multiple habitat types including sandy floodplains, open woodlands, meadows, and areas adjacent to ponds or streams, where loose soils facilitate burrowing and seasonal breeding (Yagi and Green 2012; Green et al. 2016). Further, Fowler's Toads serve as predators of many small terrestrial arthropods (Breden and Field Museum of Natural History. 1988), while also presumed to be acting as prey for birds and omnivorous small mammals such as skunks and raccoons (Barnett et al. 2023). Fowler's Toads are listed as Least Concern on the IUCN Red List due to a broad distribution, large population, and tolerance of moderately disturbed habitats (IUCN 2021a). However, localized declines have been reported in areas experiencing habitat loss, pesticide use, and competition or hybridization with sympatric toad species (Green 1984; Jones and Tupper 2015). Many amphibians face the same threats that lead to population decline as Fowlers Toads', making them a suitable model for examining conservation strategies that can then be applied to at‐risk species (59–61). Documenting how the gastrointestinal mycobiome changes in these toads from the wild to captivity will provide a translational framework for assessing the overall microbiome health of captive amphibians, informing future conservation efforts for threatened and endangered species.

In this work, we aim to perform a trial study to characterize the effects of captivity over time on the mycobiome community of a non‐threatened amphibian to inform and improve future studies on the possible effects that it may have on their hosts. To achieve this, we performed metamplicon sequencing of the subunit 1 of the Internal transcribed spacer (ITS1) region. Sequencing was performed on 53 fecal samples from fifteen wild‐caught Fowler's Toads across a four‐week timeframe (week 1: *n* = 11, week 2: *n* = 8, week 3: *n* = 9, week 4: *n* = 10), and assigned taxonomy based on Amplicon sequence variants (ASVs), a unique DNA sequence derived from amplicon sequencing data that can differ by single nucleotide changes, representing extremely fine measurements of diversity. Themycobiome diversity of these samples was then analyzed as a pooled dataset based on timeframe to characterize a “core” mycobiome or fungi that are found across all timeframes, and to document if a shift in community diversity occurs in the wild toads' mycobiome as they transition to captive environments. Using measurements of alpha and beta diversity, as well as the ecological guild to which the identified fungal taxa belong, we document a broader sense of what the general role these communities may be facilitating during these transitions in captivity. With these results, we establish a baseline understanding of the core mycobiome associated with Fowlers Toad, in addition to documenting the total community shifts that occur when these animals are transitioned from the wild to captivity, with the aim of carrying these ideas forward into future studies which may translate into usage with threatened amphibian species.

## 2 Results

### 2.1 Metamplicon Sequencing and Broad Taxonomic Assessment

The ITS1 subunit was amplified by PCR and sequenced for 53 fecal pellets (wild *n* = 15, time series; week 1: *n* = 11, week 2: *n* = 8, week 3: *n* = 9, week 4: *n* = 10). Each sample was then processed using DADA2 (Callahan et al. 2016) and assigned taxonomic identifications based on the most recent UNITE fungal reference database (Abarenkov et al. 2024). A species accumulation curve analysis was conducted to determine if sufficient sampling had occurred to account for adequate recovery of ASV diversity (Figure S2). Species‐accumulation curves for the wild isolates each captive timeframe rose rapidly but diverged and flattened markedly between wild and captive groups (Figure S2). All four captive timeframes (week 1–4) reached an asymptote between 8 and 12 samples, with their 95% confidence intervals overlapping and yielding few additional ASVs beyond that threshold. In contrast, the wild‐toad curve continued to rise even at 15 samples and showed no apparent plateau, with its 95% interval remaining constantly above those of the captive groups (Figure S2).

**FIGURE 1:**
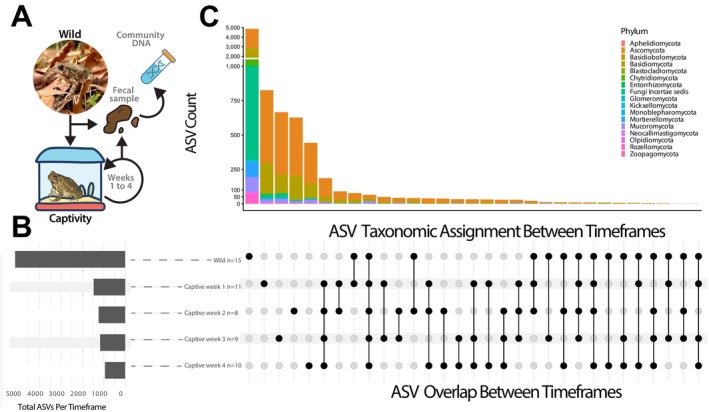
Assessing temporal dynamics of gastrointestinal fungal diversity in the wild and during captivity in Fowler's Toad. (A) Depiction of the capture of toads from wild terrestrial environments, fecal sampling, and subsequent time in captivity. (B) Total ASV count per pooled time frame. (C, Top) Stacked bar plot, with the Y‐axis representing Amplicon sequencing variant (ASV) count and the X‐axis indicating Phylum level ASV Overlap with all possible combinations of wild and captive timeframes, with color representing each fungal Phylum. Break lines have been added to the stacked bar plot for Wild samples and artificially reduced to 20% of their size to improve visualization for all other overlapping timeframes. (C, Bottom) Presence–absence dot plot where black dots mark ASV detection in each pooled timeframe and connecting lines indicate the presence of overlapping ASVs between timeframes.

Across all samples, we identified 17,771 ASVs (9,916,296 total raw reads), representing 17 phylum‐level assignments (Figure 1 and Data S1). Despite these high numbers, 9415 ASVs were assigned only to the Kingdom level Fungi (53%), indicating the sheer undescribed diversity of the amphibian gut community. At the level of phylum, we recovered 8356 ASVs, with the most numerous assigned group across all of our samples being the *Ascomycota* (*n* = 4214, 50%), followed by the *Basidiomycota* (*n* = 2508, 30%), *Mucoromycota* (*n* = 242, 3%), and all other phyla representing the remaining ASVs (*n* = 1392, 17%). All deeper classifications are further reported in an interactive table provided in the Data S1. Additionally, we also investigated broad overlap of these phylum‐level assignments. We found that the vast majority of phyla diversity was uniquely associated with the wild samples, while captive time frames were dominated by the presence of *Ascomycota* and *Basidiomycota*, with very little ASV overlap occurring between all combinations of timepoints (Figure 1C).

### 2.2 Characterization of Alpha and Beta Diversity Across Captivity

**FIGURE 2:**
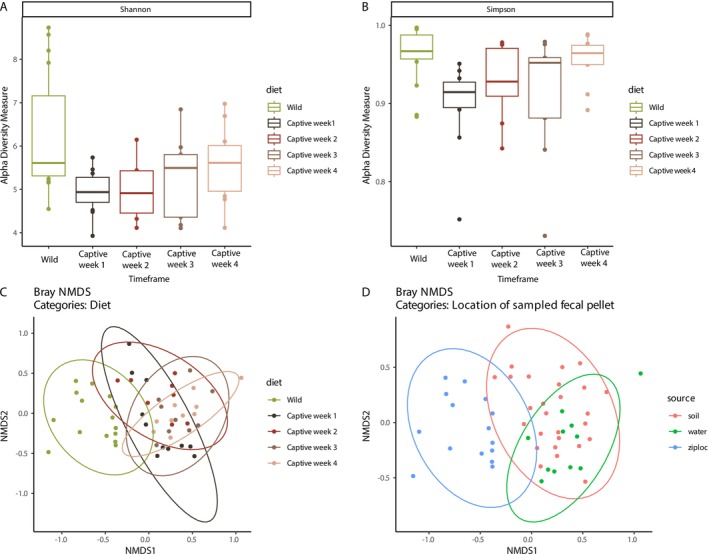
Diversity of Fowler's Toad gut mycobiomes under wild and captive conditions. (A) Shannon diversity index: Boxes show median and interquartile range, whiskers extend to 1.5× IQR, and points are individual samples. (B) Simpson's diversity index: Boxplots for the same groups and formatting as in A. (C) Bray–Curtis NMDS by time frame: Two‐dimensional ordination of all wild and captive samples, colored by time frame. Ellipses are 95% confidence intervals for each group. (D) Bray–Curtis NMDS by the physical location from which fecal pellets were collected. Ellipses are 95% confidence intervals for each group.

To investigate the overall diversity across our wild samples and captive time frames, we measure alpha and beta diversity using a Shannon (Figure 2A) and Simpson (Figure 2B) index to capture the richness and assess overly dominant taxa respectively. Metrics of alpha diversity indicate an initial, steep decline in fungal diversity during the first week in captivity with the overall diversity of the total community increasing in the following weeks. Further, we also performed Kruskal‐Wallis and pairwise Wilcoxon statistical tests to examine whether, and at what timepoints, significant differences in the alpha diversity measurements occur over time. We found that the Kruskal–Wallis test detected a significant difference in Shannon diversity among diet groups.

(*χ* ^2^ = 19.43, df = 4, *p* = 0.00065) and that wild samples have significantly higher Shannon diversity than all captive groups except 4‐week (Wild: week 1 *p* = 0.0030, Wild: week 2 *p* = 0.0168, Wild: week 3 *p* = 0.0083, Wild: week 4 *p* = 0.1819). A significant difference was also found in our measurements of Simpson diversity (*χ* ^2^ = 17.112, df = 4, *p* = 0.001838), but only showed significant differences between week 1 and week 3 compared to wild samples (Wild: week 1 *p* = 0.0055, Wild: week 2 *p* = 0.2147, Wild: week 3 *p* = 0.0224, Wild: week 4 *p* = 0.3943).

Overall, both Shannon and Simpson diversity show a significant drop immediately after animals enter captivity, remained suppressed through weeks 2–3, and show only a partial recovery by week 4, but never return to the same level of alpha diversity observed in the wild samples.

Further, we also investigated the overall similarity of the mycobiome based on diet timeframes and found statistically significant differences between wild samples and those from captivity (PERMANOVA, *F* = 3.681, *p* < 0.001, Figure 2C) and accounting for 23.5% of the overall variability of each time frame. Due to a lack of sampling and microbial community data from the wild environment and the captive environment soil and aquatic substrates, we also investigated if there were possible effects of the fecal sampling source on community diversity (Figure 2D). A Bray NMDS was also used to determine the difference between the initial wild fecal samples collected from the ziploc bags of captured toads, and the post capture fecal pellets which were sampled opportunistically from either soil or water within the toads' housing. Community composition differed significantly among sampling sources (PERMANOVA, *F* = 4.66, *p* = 0.001), with the sampling source accounting for ~15.7% of the variation in microbial community structure. The variability due to sampling location, however, was largely driven by samples derived from ziploc bags (= wild isolates), while the other substrates largely overlap (Figure 2D). This pattern is in line with other findings that this shift is likely due to the overwhelmingly higher diversity of ASVs in wild samples compared to post‐capture.

### 2.3 Temporal Shifts in the Mycobiome Community During Wild to Captive Transition, and the Core Mycobiome

**FIGURE 3:**
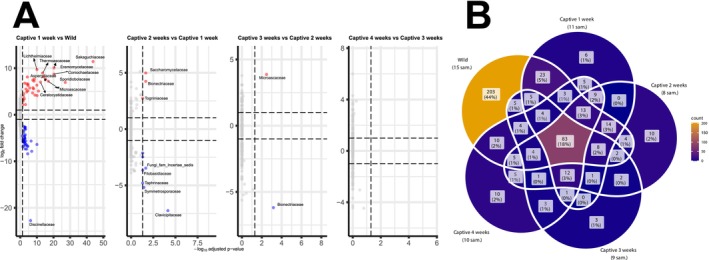
Family‐level shifts and Family‐level ASV overlap across wild and captive timeframes. (A) Volcano plots of the top 10 most significantly increased (red) and decreased (blue) amplicon sequencing variant changes by fungal Family for wild toads and weekly captive timeframes (weeks 1–4, left to right). Along the x‐axis are the −log_10_ adjusted *p*‐values; the y‐axis shows the log_2_ fold change for ASV abundance between weeks. (B) Five‐set Venn diagram of ASVs collapsed to the taxonomic level of Family shared among wild (*n* = 15) and captive toads at week 1 (*n* = 11), week 2 (*n* = 8), week 3 (*n* = 9), and week 4 (*n* = 10). Each section represents a single timeframe; the numbers within each overlapping region indicate the count of assigned families and their percentage of the total recovered ASVs.

While transitioning from the wild to captive conditions, the fungal community within the toad hosts underwent an immediate restructuring with a total of 453 ASVs differing significantly in the first week (Figure 3A). The first week of captivity exhibited the strongest shift in the mycobiome community, with many fungal families exhibiting significant changes in relative abundance, likely reflecting intermittent taxa associated with a wild‐derived diet, rather than ecological filtering, particularly for transient fungi not retained across all timeframes (Figure S3). Families such as *Lichtheimiaceae*, *Thermoascaceae*, *Bionectriaceae*, *Didymellaceae*, and *Sporidiobolaceae* exhibited sharp increases in captivity, whereas others, such as *Coniochaetaceae*, *Eremomycetaceae*, and many other *Basidiomycete* lineages, declined substantially. This initial disruption was the most dramatic, with the magnitude of changes observed in all subsequent week‐to‐week comparisons being far less substantial. From week 1 to 4, the fungal community turnover dropped sharply. Each timeframe showed comparatively fewer differentially abundant families, and the effect sizes were notably smaller than the wild‐to‐Week‐1 shift. Only a limited set of lineages, such as *Microascaceae* (Weeks 2–3) and *Bionectriaceae* (Week 1–2), displayed continued changes, indicating that most restructuring occurred immediately upon entering captivity where the mycobiome would then be representative of those picked up from the captive environment, or the “core” mycobiome of fungal taxa which are continuously present in the GI tract of Fowlers Toad.

A total of 303 ASVs across seven phyla and 83 families were found to represent the core mycobiome (e.g., species present across all stages of wilderness and captivity, Figure 3B and Data S1) within our Fowlers Toads. Wild individuals harbored the highest number of recovered fungal families (203, 44%), whereas each captive timepoint contained only a small fraction of the total unique diversity (0%–5%). The core community shared across all conditions was limited (83 families, 18% of total across all timeframes), demonstrating that most fungal taxa detected in wild toads were rapidly lost or replaced in captivity. Week‐specific ASV sets remained low throughout the experiment, reinforcing the observation that the major community shift occurred immediately and that subsequent weeks largely retained a constrained, captivity‐adapted subset of the original mycobiome.

### 2.4 Association of Fungal Trophic Guild

**FIGURE 4:**
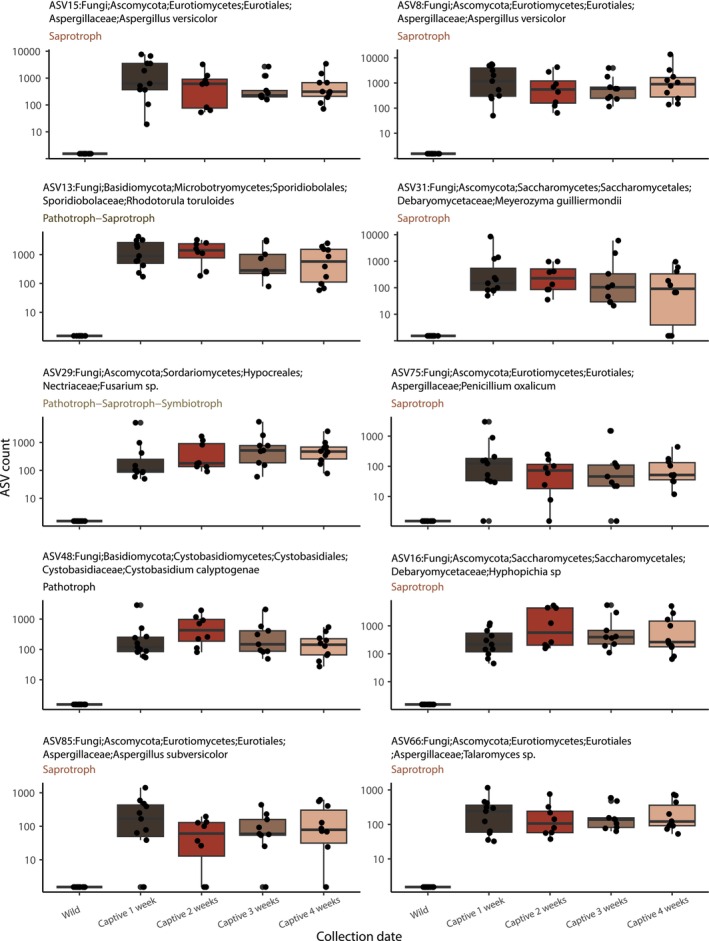
Captivity‐enriched fungal ASV dynamics. log_10_ ASV count for a subset of the ten ASVs with the largest mean increase in count between the wild and captivity. Each ASV is faceted by FUNGuild ecological guild (saprotroph, pathotroph–saprotroph, pathotroph–saprotroph–symbiotroph, pathotroph). Points represent individual samples (*n* = 9–15).

Ecological and functional guild assignments revealed clear shifts in fungal community structure between captive and wild amphibians (Figure 4 and Figure S4). In captivity, differentially abundant ASVs were dominated by saprotrophs, which accounted for the largest fraction (63 ASVs). Mixed guilds that included pathotroph–saprotroph and pathotroph–saprotroph–symbiotroph associations were present but represented far fewer ASVs (15 and 11 ASVs, respectively), and only a small number of ASVs were classified exclusively as pathotrophs (4 ASVs). Notably, no purely symbiotic guilds were detected among captivity‐enriched fungi.

In contrast, wild individuals showed a broader functional breadth of differentially abundant fungi. Nine guild categories were represented, with saprotrophs again comprising the largest group (91 ASVs), but with substantially more pathotroph–saprotroph (81 ASVs) and pathotroph–saprotroph–symbiotroph taxa (64 ASVs) compared to captivity. Additional guilds unique or nearly unique to the wild microbiome included saprotroph–symbiotrophs, pathotroph–symbiotrophs, and pure symbiotrophs, suggesting that wild amphibians harbor fungal assemblages with more complex ecological strategies and potentially greater ecological connectivity.

Together, these patterns indicate that captivity reduces both the diversity of functional guilds and the representation of symbiotic fungi, yielding a more simplified community dominated by saprotrophic taxa. Conversely, wild individuals maintain a more functionally diverse fungal consortium that includes a wider array of ecological roles and interactions.

## 3 Discussion

Our study examined the core microbiome and the dynamic community shifts that occur in the common Fowler's toad, and we documented how captivity impacts fungal diversity, taxonomic composition, and functional guilds associated with Fowler's toad mycobiomes. Although bacterial microbiomes have historically received more attention, fungal communities play an important role in environmental and host‐associated settings (McKnight et al. 2022). A growing body of work shows that fungal communities also contribute distinct and complementary functions, including adaptations specifically to the herptile GI environment (Vargas‐Gastélum et al. 2024) and the balance of bacterial‐fungal interactions, which can lead to skin disorders (Nguyen and Kalan 2022), respectively. However, current evidence does not suggest that fungi are more or less important than bacteria; rather, both groups are likely to be complementary and even share functions within the environment (McKnight et al. 2022). We chose to focus on the mycobiome of the GI due to its relatively unknown and understudied nature within Fowler's toad and in amphibians broadly.

This work was performed as a trial to characterize if there are drastic effects on the mycobiome associated with transition to captivity, to inform future husbandry practices of other amphibians. Given the increasing extinction pression on amphibians, a better understanding of the interplay between captive and wild environment may lead to more successful capture and release breeding programs, particularly for amphibians which are considered threatened or endangered. Below we discuss the roles the mycobiome may be playing in the health of the host, how these shifts in community could be detrimental to long‐term captive host health, and the perspective and future work that could be done to refine these studies for applicability to a broader array of amphibians.

Our results show that the mycobiome community within Fowler's toad undergoes dramatic restructuring upon being moved to captivity. In our analysis, each ASV is treated as a distinct ‘nominal taxon.’ As seen in Figure 4, multiple ASVs (e.g., ASV8 and ASV15) may be assigned to the same binomial species, such as Aspergillus versicolor. This reflects intra‐genomic variation or the presence of multiple strains within the host, providing a higher resolution of community restructuring than species‐level clustering alone. Both Shannon and Simpson diversity metrics demonstrate a statistically significant collapse in diversity during the first week after animals are brought into captivity, indicating the simultaneous loss of rare taxa and the emergence of less diverse communities dominated by only a few fungal lineages. Although total diversity increases modestly through weeks 2–4, this rebound is incomplete: by the end of the four‐week period, neither richness nor evenness returns to levels observed in wild individuals. This pattern is consistent across statistical tests. Shannon diversity differs significantly among timepoints, with wild individuals exhibiting higher diversity than weeks 1–3, and Simpson diversity shows a similar but slightly weaker trend, reflecting the disproportionate loss of rarer taxa in early captivity.

The initial “crash” collectively indicates that captivity induces both an immediate and persistent contraction of the GI associated mycobiome. While speculative, these early losses are likely due to the loss of transient mycobiome members associated with a wild diet and environmental landscape. In particular, wild Fowlers Toad are carnivorous with a diet composed primarily of insects (particularly beetles) and gastropods (Walton 1988). In contrast, captivity diets consist of commercially sourced crickets and beetle larvae, which themselves are raised in non‐wild conditions, and thus would likely have a reduced natural microbial community associated with them. This pattern can further be extended to the housing conditions as well. Wild toads are primarily terrestrial and move through leaf litter and detritus, where they are likely to also pick up transient members of the soil microbial community, while in captivity this is reduced to cleaned and treated water and a solid substrate made of processed coconut fiber. Taken together, the pattern of extreme total diversity loss upon initial movement to captivity is likely to be driven by the loss of fungal taxa transiently associated with the host's wild diet and environment upon capture.

After the initial diversity crash, a partial recovery over the subsequent 4 weeks occurred. *Ascomycota* remained the most prevalent fungal group across all conditions, though their relative abundance increased over time in captivity. *Basidiobolomycota* showed a steep decline from 37% in wild conditions to just 5% by the fourth week of captivity. This suggests that non‐transient *Basidiobolomycota* are sensitive to environmental changes associated with captivity. In particular, some taxa such as *Didymellaceae* and *Bionectriaceae* are commonly found in soil and insect frass and are likely to be found in greater numbers with feeder insects, while *Lichtheimiaceae, Thermoascaceae and Sporidiobolaceae* thrive on decomposing chitin‐rich insect substrates (Vega and Blackwell 2005; Houbraken et al. 2020). These increases indicate a shifting to a microbiome community directly associated with an artificial diet, along with opportunistic colonization in response to the novel captive environment, followed by competitive exclusion by other taxa. Microbial shifts such as these have been well documented in non‐amphibian captive populations of host animals, including birds such as the Brown Kiwi (San Juan et al. 2021), primates (Wang et al. 2021; Suzuki et al. 2025), and large mammals like the black rhinoceros (Gibson et al. 2019), supporting an overall pattern of microbial communities becoming heavily influenced by captive conditions.

Despite this growing body of literature, many significant gaps remain in our understanding of how captivity influences microbial communities in less‐studied taxa, such as amphibians. While the amphibian gut mycobiome remains poorly characterized, studies on the skin microbiome have occurred and show marked shifts in community composition and diversity following captivity, even characterizing a partially restored microbiome and increased antifungal properties upon controlled release of the harlequin frog, *Atelopus varius* . Additionally, other studies have investigated how both environmental context and interspecific interactions can alter the composition and stability of amphibian microbiomes. Medina et al. (2021) demonstrated that co‐infecting pathogens create an additive effect on host microbiomes, showing that pathogen pressure can alter the microbial community within external and internal niches. Similarly, Greenspan et al. (2019) showed that arthropod and bacteria interactions influence the microbiomes of aquatic hosts and pathogen defense, emphasizing that biotic factors, especially those affecting the skin associated community, can have dramatic effects. Similar to these previous studies, our gut‐associated communities exhibit reductions in diversity and altered taxonomic structure under captive conditions, suggesting comparable environmental filtering effects. However, the magnitude and direction of these changes are likely to differ between skin and gut habitats, reflecting their distinct ecological roles and microbial exposure.

An important first step in addressing the importance of contribution of the GI‐associated mycobiome to its host is to define its “core” mycobiome, and what its potential role in the overall host environment may be. Here, we found that 308 ASVs (from 83 families) could be recovered in all wild and captive timeframes, and thus comprised the core community that are likely to be associated with Fowlers Toad either as consistent mycoflora, or those which are likely to be consistently found in association within their diet and habitat, regardless of wild or artificial settings. Our core mycobiome was dominated by families including *Basidiobolaceae* (known GI‐associated symbionts of amphibians), the *Mucoraceae*, (fast growing and often associated with detritus and decaying insect matter) (Mendoza et al. 2015), and *Aspergillaceae* (Data S1). Investigating further, *Basidiobolus* species form symbiotic relationships with amphibians and have been consistently found across different geographic locations and hosts (Walker et al. 2020; Vargas‐Gastélum et al. 2024; Hincher et al. 2025), although the role they play as symbionts remains unknown. Likewise, core *Aspergillaceae*, such as *Aspergillus*, are near ubiquitous fungi found indoor and renowned for their carbohydrate‐degrading enzymes (de Vries and Visser 2001), which may complement host digestion, or act as an opportunistic pathogen (Seyedmousavi et al. 2015). Meanwhile, the rapid growth of *Mucoraceae* under fluctuating moisture hints at its resilience to substrate shifts in captivity. These conserved assemblages could underpin essential digestive, immunological, and perhaps even behavioral functions, providing a microbial “backbone” that persists despite the perturbations of artificial environments, which has been shown in other model organisms such as mice (Hanski et al. 2024) and endangered species, such as the Giant Panda (Huang et al. 2023). By characterizing these steadfast fungal partners, we establish a baseline against which the health and stability of amphibian mycobiomes can be assessed in both conservation and husbandry contexts.

Due to the relatively cryptic nature and understanding of many of these fungi, we also sought to assign the ecological guilds with FUNGuild (Nguyen et al. 2016) to understand broader patterns that were prevalent. Function guild assessment mirrored our diversity results with a stark functional restructuring of the toad mycobiome under captivity. Among ASVs enriched in captivity, saprotrophs dominated with 63 ASVs, followed by pathotroph–saprotrophs (15 ASVs), pathotroph–saprotroph–symbiotrophs (11 ASVs), and pure pathotrophs (4 ASVs). In contrast, ASVs that declined from the wild encompassed nine guilds, led by saprotrophs (91 ASVs), pathotroph–saprotrophs (81 ASVs), and pathotroph–saprotroph–symbiotrophs (64 ASVs), but also including saprotroph–symbiotrophs (30 ASVs), pure pathotrophs (17 ASVs), and even rare pure symbiotrophs (2 ASVs). *Mucoromycota*, which contains microfungi with broad ecological guilds, including saprophytic and pathotrophic, showed a steady increase over time. The noticeable shift of ASVs assigned to *Mucoromycota* with FUNGuild assessments of Saprotroph (*n* = 231/242) versus Saprotroph‐Symbiotroph (*n* = 11/242) suggests that captivity provides conditions favorable for this group, possibly through shifts in moisture levels or substrate availability, or through an increase in niche space, such as the availability of nutrients that are no longer being actively degraded by transient fungi from the wild. Notably, no ASVs classified solely as symbiotrophs were differentially abundant between nature and captivity, indicating there is little difference in the abundance of dedicated mutualists under both natural and *ex situ* conditions. Alternatively, a strong pattern of guild plasticity within symbiotrophs is presented from our data. The shift toward saprotrophic and pathotrophic lifestyles, and away from symbiotic modes, suggests that dietary simplification and altered substrates in captivity favor generalist decomposers, potentially at the expense of fungi that provide host‐beneficial services such as nutrient provisioning or pathogen defense. Maintaining functional guild diversity, therefore, may be as critical as preserving taxonomic diversity in captive husbandry and reintroduction programs.

### 3.1 Perspectives, and Future Considerations in Understanding the Shifts in Mycobiome Composition as Part of Endangered Species Conservation Efforts

Building on our trial data, we can leverage the temporal patterns of diversity loss and taxonomic turnover we observed in Fowler's Toads to investigate “microbial crashes” in other at‐risk amphibians.

However, several caveats should be considered when interpreting these results. First, our study pooled samples rather than tracking individual toads over time, which allowed us to capture a broad pattern of mycobiome community dynamics, but lost individual‐level resolution. Further, the absence of environmental sampling including wild substrate, captive substrate (e.g., coconut shavings), enclosure water, and food item, means we cannot directly distinguish whether transient fungal taxa originated from ingestion or environmental sources. As a result, some fungal “disappearance” or “reappearance” events likely reflect intermittent exposure rather than biological filtering. Additionally, natural diets of wild Fowler's toads, which include a broad assemblage of arthropods and gastropods, differ substantially from the simplified diet provided in captivity. Our results show that captivity rapidly alters the gut mycobiome, driven largely by shifts in diet and husbandry. This implies that conservation programs should mimic natural conditions as closely as feasible, specifically in trying to recreate the wild host diet, to retain a more representative gut community. While biosecure facilities (such as Zoo's and research facilities) cannot fully recreate native environments, even partial alignment may help reduce early‐onset microbial dysbiosis before release. Further still, animals which are captured from different localities along the range of the Fowlers Toads are likely to have geographic specific fungal lineages associated with their mycobiome as well.

These limitations emphasize the complex and difficult nature of conducting these studies but also highlight just how important the environment is to the microbial communities associated with any host organism. While the microbiome members reported here are specific to Fowler's Toad, the overall patterns of crash, recovery, and shift to in situ associated community structure could help to inform future captivity studies of endangered amphibians. Fowler's Toads are abundant and widespread, making them a suitable model for examining conservation strategies that can then be applied to at‐risk species (Poo and Hinkson 2020; Poo et al. 2022; Malter et al. 2025).

Our framework can be applied to critically endangered species that rely heavily on captive‐release programs, such as the Wyoming Toad ( *Anaxyrus baxteri* ) and dusky gopher frogs ( *Lithobates sevosus* ). In these cases, captive‐bred individuals that are naive to the complexity of their natural habitats are released into the field to build new wild populations (Odum 2005; Bogisich et al. 2025). Monitoring shifts in mycobiome structure across life stages and release sites could pinpoint fungal partners essential for larval and juvenile development and pathogen defense, informing husbandry adjustments (e.g., substrate choices or probiotic applications) that preserve functional guilds. Similarly, we can extend this to other amphibians such as eastern Hellbender salamanders ( *Cryptobranchus alleganiensis* ). Hellbender salamanders are one of North America's largest amphibians and face dire population declines across much of their range due to habitat loss, water pollution, disease, and stream sedimentation. Although extant in distinct populations, they are considered near threatened or endangered in many states (IUCN 2021b). By sampling hellbenders at key stages of head‐starting and captive‐breeding programs, we can test whether similar rapid losses of fungal diversity occur upon transfer to artificial stream enclosures and whether particular taxa (e.g., core *Ascomycetes* or *Basidiobolales*) resist collapse or predict reintroduction success. Ultimately, transferring our insights from Fowler's Toads to these conservation and species recovery programs will help identify microbial indicators of host health, optimize *ex situ* rearing conditions, and improve survival rates upon release, which contribute toward mitigating amphibian declines worldwide.

## 4 Methods

### 4.1 Animal Capture and Care

Animal handling and care procedures were approved by the Memphis Zoo Institutional Animal Care and Use Committee (Approval 2022‐04) and the Tennessee Wildlife Resource Agency (Permit 2430), which are described briefly below. In May 2022, fifteen adult Fowler's Toads were captured by hand in relatively even sex ratios (Female = 7, Male = 6, Unknown = 2) in Shelby County, Tennessee (USA). Toads were located between 2100 and 2400 h through visual encounter surveys using a headlamp with a brightness of 1250 lm. The sex of captured toads was determined by the presence of a darker throat in males, a secondary sexual characteristic present in males during the breeding season. Toads were housed in same‐sex groups, to avoid breeding in captivity, of four individuals in 10‐gal glass aquariums (50.8 cm L × 25.4 cm W × 30.48 cm H) outfitted with commercial coconut shavings (Zoo Med Eco Earth Compressed Coconut), tap water that was dechlorinated and conditioned using API Tap Water Conditioner, and a hide box for cover. Daily water changes were conducted, and spot cleaning of excrement was performed, with substrate replacement occurring as needed. The provided diet consisted of a combination of commercially sourced crickets (dusted with calcium powder), mealworms, and superworms offered *ad libitum* four times per week. Artificial light was provided in a 12:12 h light‐to‐dark cycle, and the room was maintained at ~23°C. All materials and tools used during the study were cleaned with a diluted 15% bleach solution and thoroughly rinsed with tap water before use.

### 4.2 Fecal Collections, DNA Extraction, Quantification, and Control

Upon capture, toads were placed individually in a large ziploc bag (*n* = 15) where the first fecal sample was collected in a single evening before being exposed to captive environments or non‐natural food sources. After the initial wild diet fecal samples were collected, fresh fecal pellets (deposited the day of) were opportunistically sampled from singular toads (the weekly fecal collection) from each enclosure every 7 days for 4 weeks, but samples from distinct individual toads were not tracked from week to week. This resulted in 11 fecal pellets from week 1, 8 from week 2, 9 from week 3, and 10 from week 4, for a total of 53 samples for sequencing. A single fecal pellet was scooped into and placed into its own 2 mL Eppendorf tube and stored frozen at −20°C until ready for processing. Crucially, pellets were not physically macerated or pooled together prior to DNA extraction. Total gDNA was extracted using the QiAmp PowerSoil Pro DNA Kit (Qiagen, Cat. No. 51804) using the manufacturer's suggested protocol with the specific modifications of initial vortexing time of the fecal sample increased to 15 min to ensure homogeneity with the C1 (lysis) buffer, and the C3 (wash) and C6 (elution) buffers were incubated at 65°C before use in a water bath. DNA concentrations for each sample were measured with a Qubit 4 Fluorometer using the 1× dsDNA High Sensitivity reagents (ThermoFisher Scientific). Sample purity was also checked at 260, 280, and 230 nm with a Nanodrop One Microvolume UV–Vis Spectrophotometer (ThermoFisher Scientific), and only samples that met sequencing thresholds of more than 200 ng of total DNA and optical density 260/280 of 1.8–2.0 were submitted for sequencing. All gDNA extracts were stored at −20°C until they were ready for metabarcode sequencing.

### 4.3 Metabarcode Sequencing and Taxonomic Classification

To characterize the microbial community, we generated metamplicon data of the ITS1 subregion of the fungal internal transcribed spacer region, considered one of the most reliable housekeeping genes for fungal species identification (Schoch et al. 2012). ITS1 was chosen over the second subunit (ITS2) due to its tendency to recover higher levels of diversity in non‐dikarya microfungal phyla (Blaalid et al. 2013). The first round of amplification included three negative controls containing only water, and metabarcoding amplification was performed for each sample using the ITS5‐1737F (5′‐GGAAGTAAAAGTCGTAACAAGG‐3′) and ITS2‐2043R primers (5′‐GCTGCGTTCTTCATCGATGC‐3′) by NovoGene Inc. (Sacramento, CA), who also performed the sequencing library preparation. Negative controls produced no sequencing data and were excluded in downstream analysis. Pooled samples were then sequenced using PE 2X250 Illumina libraries on a Novaseq 6000 platform. Raw sequencing statistics are reported in Table S1. Demultiplexing was also performed at Novogene using their in‐house bioinformatic service. Raw reads were processed in R using sequence trimming specific to the variable ITS region using cutadapt v3.4 (Martin 2011), and the DADA2 pipeline to produce amplicon sequence variants (ASVs) with standard settings (Callahan et al. 2016). Taxonomic classification of ASVs was performed with the UNITE database (sh_general_release_dynamic_04.04.2024) (Nilsson et al. 2019; Abarenkov et al. 2024) before combining all outputs into a Phyloseq (McMurdie and Holmes 2013) object for downstream analysis, where ASV is defined as a unique DNA sequence that differ by a single nucleotide.

### 4.4 Data Exploration and Visualization

Data exploration, visualization, and generation of interactive ASV tables (Data S1) were performed using the R package MiscMetabar v0.14.2 (Taudière 2023). Preprocessing of our phyloseq object was first performed using the function clean_pq with simplify_taxo = TRUE to clean and simplify the taxonomic classifications within our OTU matrix. All downstream analysis was performed by subsectioning our dataset into the five individual timepoints that fecal samples were collected in. An overall visual summary for our phyloseq object was created using the summary_plot_pq function (Figure S1). To generate the UpSet plot (Figure 1) to investigate phyla overlap between diet time frames, we first subset our phyloseq object using the ps_clean function from MiscMetabar at the Phylum level, removing all NA designations. The upset_pq of MiscMetabar was then used with a minimum number of sequences set to 2 to remove single ASVs. Volcano plot analysis (Figure 3, Left) was performed using the R package EnhancedVolcano v. 1.29.1 (Blighe 2026). To visualize the overlap of Family‐level ASVs across all diet time frames, a Venn diagram (Figure 3, Right) was generated with the ggvenn_pq function of MiscMetabar with the options taxonomic_rank “Family”.

### 4.5 Species Accumulation Analysis

A species accumulation plot was generated from our phyloseq object using the specaccum function of the R package VEGAN v2.6–10, a suite of programs and functions designed for community ecology, using a random sampling method with 100 permutations (Dixon 2003). Samples were grouped by diet time frames, and 95% ASV recovery thresholds were quantified and plotted using ggplot2 v3.5.2 (Wickham 2016) (Figure S2).

### 4.6 Statistical and Biodiversity Measurements and Fungal Trophic Guild Determination

Community analyses were performed using the R package Phyloseq V1.44.0. Visualizations, including graphs and figures, were plotted using the data visualization package ggplot2, a comprehensive suite of functions for interfacing with graphics in R. The Shannon diversity index, used to assess alpha diversity, and the Bray–Curtis dissimilarity index, used to evaluate beta diversity, were employed to analyze differences in community composition. This approach considered relative abundances, ignored shared absences, and effectively captured ecological gradients, making it ideal for analyzing microbial community shifts across our treatments. The identification of differentially abundant taxa before and after captivity was determined using DESeq2 (Love et al. 2014). The variance‐stabilizing transformation (VST) was used to normalize count data, and differentially abundant taxa were identified with the DESeq function (adjusted *p*‐value < 0.01). log2 fold changes were calculated for significant taxa enrichment or depletion in fecal samples collected before placement in captivity and at each subsequent week. A quality control cutoff was introduced to reduce noise in the dataset by only including taxa if a log_2_‐foldchange in abundance greater than two and an adjusted *p*‐value of 0.01 was observed. Taxa that could not be assigned (NA) at the phylum level were excluded from downstream analysis as they provided no distinct information to be evaluated. In addition to the taxonomic assignment, the ecological guild, which has been shown to provide a broad estimation of what a fungal community may be doing within an environment, was assigned to ASVs using the FUNGuild database v1.1. Significant differences in trophic mode abundance were analyzed by comparing wild samples to all captivity weeks and vice versa and plotted using ggplot2.

## Author Contributions

**Alexander J. Bradshaw:** data curation (equal), formal analysis (equal), methodology (equal), visualization (equal), writing – original draft (equal), writing – review and editing (equal). **Sinlan Poo:** conceptualization (equal), funding acquisition (equal), investigation (equal), methodology (equal), project administration (equal), resources (equal), supervision (equal), visualization (equal), writing – original draft (equal), writing – review and editing (equal). **Tracey E. Malter:** conceptualization (equal), investigation (equal), methodology (equal), project administration (equal), writing – review and editing (equal). **Rylie M. Strasbaugh:** data curation (equal), investigation (equal), methodology (equal), project administration (equal). **Brianna Bodner:** data curation (equal), investigation (equal), methodology (equal), project administration (equal). **Madison R. Hincher:** formal analysis (equal), investigation (equal), methodology (equal). **Anne Devan‐Song:** conceptualization (equal), investigation (equal), methodology (equal), project administration (equal), supervision (equal), writing – original draft (equal), writing – review and editing (equal). **Javier F. Tabima:** conceptualization (equal), data curation (equal), formal analysis (equal), methodology (equal), visualization (equal), writing – original draft (equal), writing – review and editing (equal).

## Conflicts of Interest

The authors declare no conflicts of interest.

## Supporting information


**Table S1:** Sample information and sequencing statistics Fecal sampline information including the timepoint of sampling, the number of sequenced reads, the source of the pellet collection, sex of the toad and NCBI accession numbers.

**Figure S1:** Visual summary of Phyloseq object statistics. Visual summary of the phyloseq object representing all raw data from this work. Separate data matrices are represented by color squares and labeled with their designation within the phyloseq object. Primary stats are reported for the total phyloseq object adjacent to @sam_data where the acronym Nb refers to “number”.
**Figure S2:** Species‐accumulation curves of gut fungal ASV richness in Fowler's Toads under wild and captive conditions. Mean cumulative ASV richness (y‐axis) is plotted against the number of fecal samples (x‐axis) for wild toads and individuals in captivity. Shaded bands represent 95% confidence intervals around each curve. Vertical lines indicate the sample size at which each group reaches 95% of its asymptotic richness. Wild toads display a continuously rising curve without a plateau, whereas all captive groups plateau by 8–12 samples.
**Figure S3:** Volcano plots of the top ten most significant amplicon sequencing variant changes by fungal Family for wild toads compared to each weekly captive timeframe (week 1–4, left to right). The x‐axis is indicates the −log_10_ adjusted *p*‐values for changes; the y‐axis shows the log_2_ fold change for ASV abundance between the wild and each week. Samples in red represent an increases in abundance, while blue denotes a decrease in abundance.
**Figure S4:** Captivity‐enriched fungal ASV dynamics. log_10_ ASV count for a subset of the ten ASVs with the largest mean decrease under captivity faceted by FUNGuild ecological guild (saprotroph, pathotroph–saprotroph, pathotroph–saprotroph–symbiotroph, pathotroph). Points represent individual samples.

## Data Availability

All raw sequencing data have been deposited in the Short Read Archive (SRA) under Bioproject number PRJNA1266671, including Individual SRA and Biosamples (Table S1). All supplementary data, including the full Rmd file and phyloseq objects, as well as an interactive ASV table, can be downloaded from the Open Science Framework (OSF) under DOI: 10.17605/OSF.IO/5ANZ2. Any code or specific script requests should be sent to the corresponding author.
