# Supplementary material for: Characterization of a Core Fungal Community and Captivity‐Induced Gut “Mycobiome” Change in Fowler's Toad ( Anaxyrus fowleri )
Source: Ecol Evol. 2026 Apr 8;16(4):e73430. doi: 10.1002/ece3.73430 (PMC13061749; doi:10.1002/ece3.73430)

**Supplementary Figures**

**S1: Visual summary of Phyloseq object statistics**

Visual summary of the phyloseq object representing all raw data from this work. Separate data matrices are represented by color squares and labeled with their designation within the phyloseq object. Primary stats are reported for the total phyloseq object adjacent to @sam_data where the acronym Nb refers to “number”.


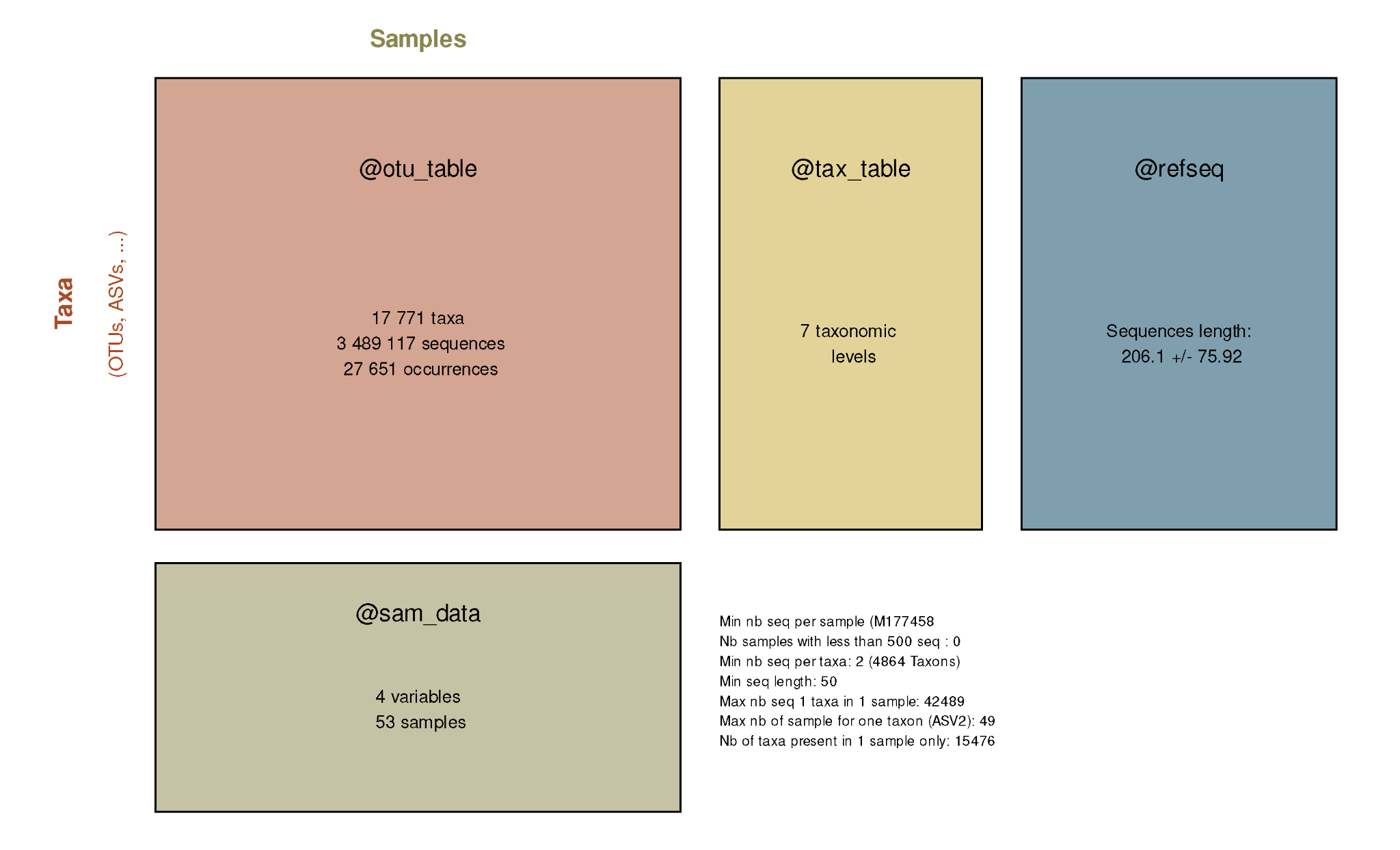


S2: **Species‐accumulation curves of gut fungal ASV richness in Fowler’s Toads under wild and captive conditions.**

Mean cumulative ASV richness (y-axis) is plotted against the number of fecal samples (x-axis) for wild toads and individuals in captivity. Shaded bands represent 95 % confidence intervals around each curve. Vertical lines indicate the sample size at which each group reaches 95 % of its asymptotic richness. Wild toads display a continuously rising curve without a plateau, whereas all captive groups plateau by 8–12 samples.


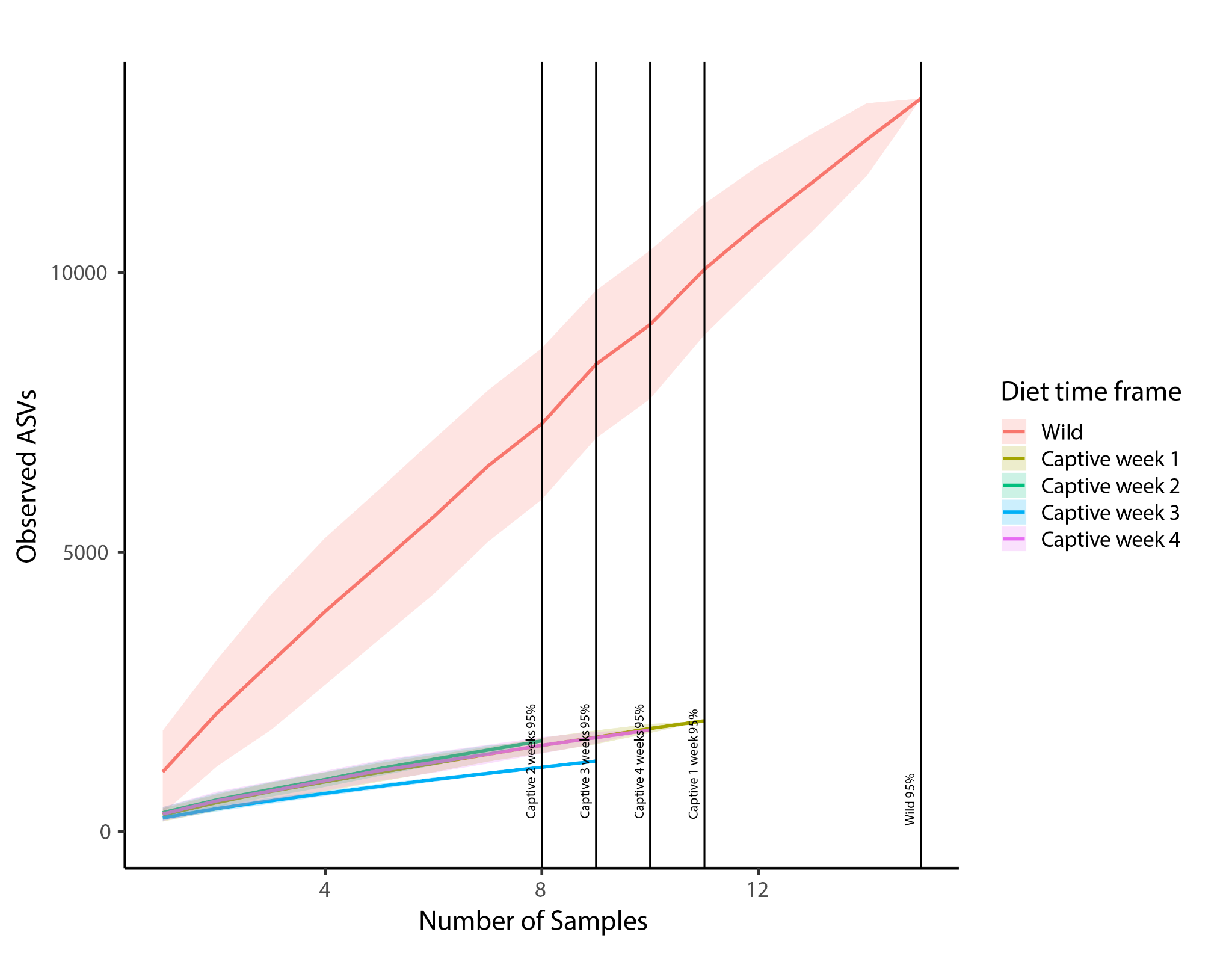


S3:

Volcano plots of the top ten most significant amplicon sequencing variant changes by fungal Family for wild toads compared to each weekly captive timeframe (wk 1–4, left to right). The x-axis is indicates the -log_10_ adjusted p-values for changes; the y-axis shows the log_2_ fold change for ASV abundance between the wild and each week. Samples in red represent an increases in abundance, while blue denotes a decrease in abundance.


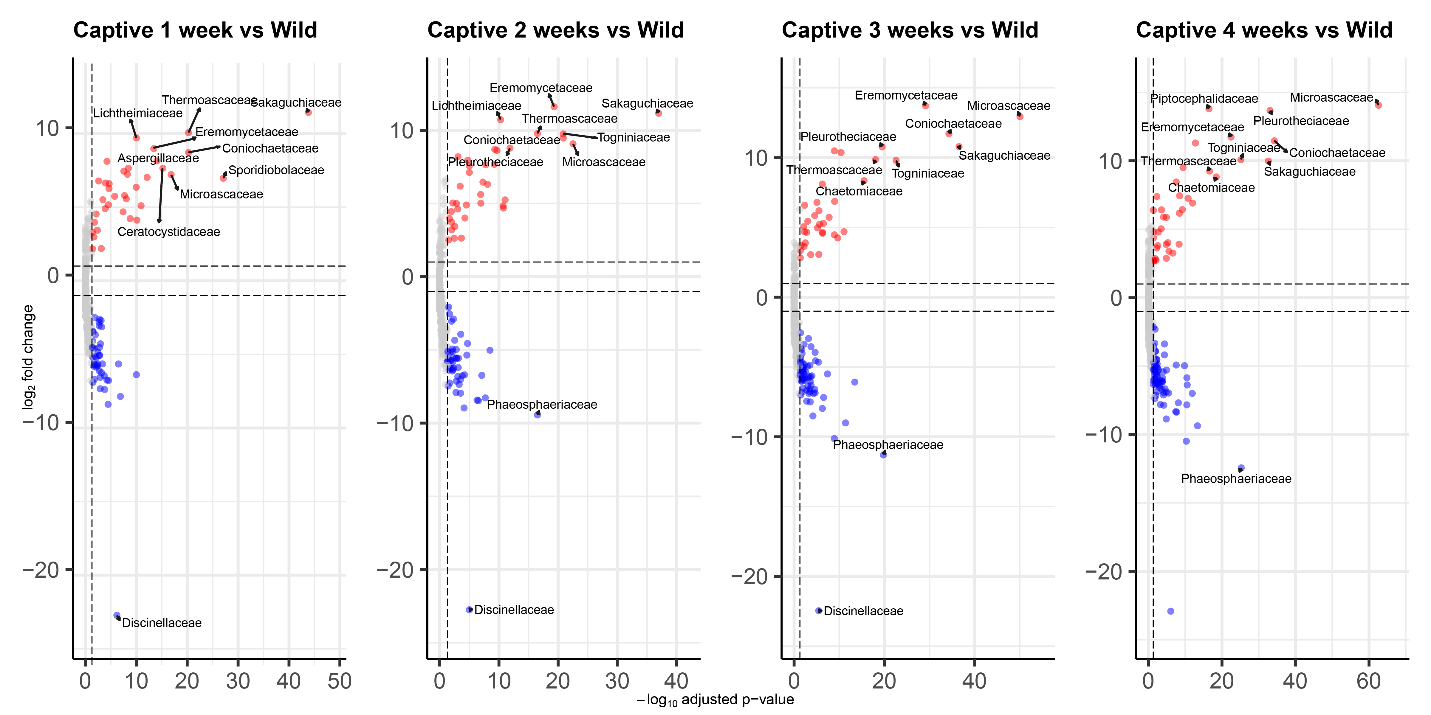


S4. **Captivity‐enriched fungal ASV dynamics.** Log₁₀ ASV count for a subset of the ten ASVs with the largest mean decrease under captivity faceted by FUNGuild ecological guild (saprotroph, pathotroph–saprotroph, pathotroph–saprotroph–symbiotroph, pathotroph). Points represent individual samples.


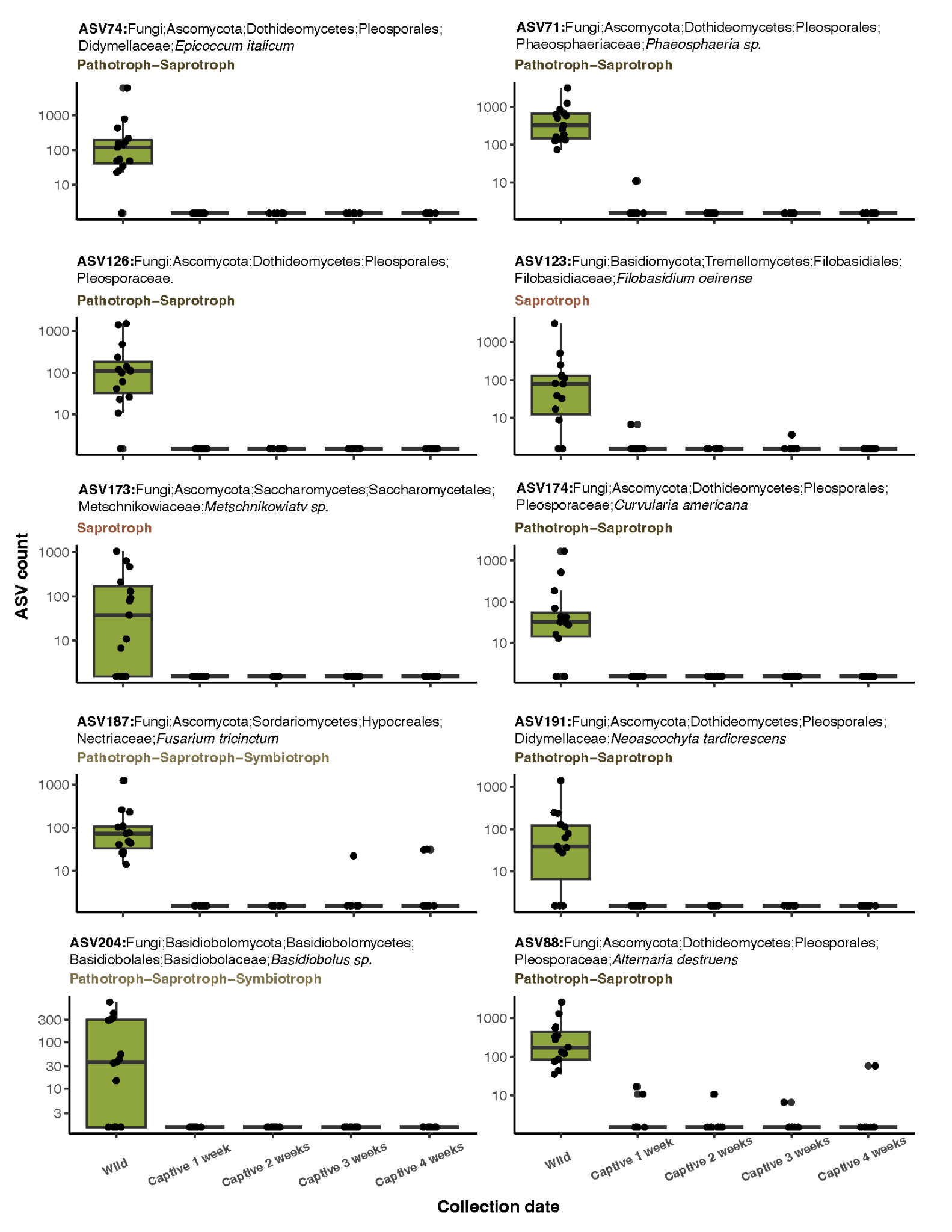

Supplement: Supplementary file 2 — Figure S1: Visual summary of Phyloseq object statistics. Visual summary of the phyloseq object representing all raw data from this work. Separate data matrices are represented by color squares and labeled with their designation within the phyloseq object. Primary stats are reported for the total phyloseq object adjacent to @sam_data where the acronym Nb refers to “number”. Figure S2: Species‐accumulation curves of gut fungal ASV richness in Fowler's Toads under wild and captive conditions. Mean cumulative ASV richness (y‐axis) is plotted against the number of fecal samples (x‐axis) for wild toads and individuals in captivity. Shaded bands represent 95% confidence intervals around each curve. Vertical lines indicate the sample size at which each group reaches 95% of its asymptotic richness. Wild toads display a continuously rising curve without a plateau, whereas all captive groups plateau by 8–12 samples. Figure S3: Volcano plots of the top ten most significant amplicon sequencing variant changes by fungal Family for wild toads compared to each weekly captive timeframe (week 1–4, left to right). The x‐axis is indicates the −log10 adjusted p‐values for changes; the y‐axis shows the log2 fold change for ASV abundance between the wild and each week. Samples in red represent an increases in abundance, while blue denotes a decrease in abundance. Figure S4: Captivity‐enriched fungal ASV dynamics. log10 ASV count for a subset of the ten ASVs with the largest mean decrease under captivity faceted by FUNGuild ecological guild (saprotroph, pathotroph–saprotroph, pathotroph–saprotroph–symbiotroph, pathotroph). Points represent individual samples. [file ECE3-16-e73430-s002.docx]
